# Irrelevant speech impairs serial recall of verbal but not spatial items in children and adults

**DOI:** 10.3758/s13421-022-01359-2

**Published:** 2022-10-03

**Authors:** Larissa Leist, Thomas Lachmann, Sabine J. Schlittmeier, Markus Georgi, Maria Klatte

**Affiliations:** 1 Cognitive and Developmental Psychology, University of Kaiserslautern, Erwin-Schrödinger-Str. 57, 67663 Kaiserslautern, Germany; 2 Centro de Investigación Nebrija en Cognición, Facultad de Lenguas y Educacion, Universidad Nebrija, Madrid, Spain; 3 Teaching and Research Area of Work and Engineering Psychology, RWTH Aachen University, Aachen, Germany

**Keywords:** Visuo-spatial short-term memory, Verbal short-term memory, Serial recall, Irrelevant sound effect, Auditory distraction, Changing-state effect, Children, Attention

## Abstract

Immediate serial recall of visually presented items is reliably impaired by task-irrelevant speech that the participants are instructed to ignore (“irrelevant speech effect,” ISE). The ISE is stronger with changing speech tokens (words or syllables) when compared to repetitions of single tokens (“changing-state effect,” CSE). These phenomena have been attributed to sound-induced diversions of attention away from the focal task (attention capture account), or to specific interference of obligatory, involuntary sound processing with either the integrity of phonological traces in a phonological short-term store (phonological loop account), or the efficiency of a domain-general rehearsal process employed for serial order retention (changing-state account). Aiming to further explore the role of attention, phonological coding, and serial order retention in the ISE, we analyzed the effects of steady-state and changing-state speech on serial order reconstruction of visually presented verbal and spatial items in children (n = 81) and adults (n = 80). In the verbal task, both age groups performed worse with changing-state speech (sequences of different syllables) when compared with steady-state speech (one syllable repeated) and silence. Children were more impaired than adults by both speech sounds. In the spatial task, no disruptive effect of irrelevant speech was found in either group. These results indicate that irrelevant speech evokes similarity-based interference, and thus pose difficulties for the attention-capture and the changing-state account of the ISE.

## Introduction

In everyday life, cognitive tasks are often performed in the presence of irrelevant background sounds such as speech, traffic noise, or music. It is therefore important to know whether and how cognitive processes are affected by task-irrelevant environmental sounds in order to avoid or attenuate possible strain and performance impairments. Consequently, numerous studies have been conducted over the recent decades to examine the characteristics of sounds, tasks, and the individuals exposed that determine the effects of sounds on mental performance (for reviews, see Klatte et al., 2013; Schlittmeier & Marsh, 2021; Szalma & Hancock, 2011).

The current study focuses on the cross-modal effects of irrelevant sounds, i.e., effects of irrelevant sound on tasks that require processing of visually presented information. According to the *duplex-mechanism framework* proposed by Hughes et al. (2007), such effects may result from two separable mechanisms. On the one hand, attention may be diverted away from the focal task and towards the sound (attentional capture). A well-studied manifestation of sound-induced attention capture is the auditory deviation effect, i.e., the decrement in visual task performance due to an unexpected change in the auditory environment (e.g., a spoken letter A embedded in a sequence of Bs: BBBBB**A**BB). Detrimental effects of auditory deviants have been demonstrated across a range of cognitive tasks, indicating that the auditory deviation effect is not dependent on specific task requirements (for reviews, see Hughes, 2014; Vachon et al., 2017). On the other hand, sound-induced performance decrements may result from direct interference between specific processes involved in the automatic, obligatory processing of the irrelevant sound, and deliberate processes involved in the execution of the focal task. Specific interference is expected to occur whenever obligatory sound processing depletes processing resources that are relevant for the current task.

Cross-modal effects of irrelevant sounds have been intensively studied in the context of verbal short-term memory. The task used in these studies requires immediate serial recall of sequences of five to nine unrelated items (e.g., digits, letters, words) presented one-by-one on a screen, with a rate of one to two items per second. Performance is appreciably impaired by task-irrelevant speech that the participants are instructed to ignore. This so-called “irrelevant speech effect” (ISE) is reliable even with low-intensity speech sounds (Colle, 1980; Ellermeier & Hellbrück, 1998), and when sound presentation is confined to a rehearsal phase after encoding of the list items (Elliott et al., 2016; Macken, Mosdell, & Jones, 1999b).

Since its discovery by Colle & Welsh, 1976, the ISE has been considered primarily as a manifestation of specific interference. Early studies conducted within the framework of Baddeley´s multi-component model of working memory (WM) focused on speech as irrelevant sound and attributed its disruptive effect on verbal short-term memory to interference between phonological representations of visual and auditory origin in the phonological loop (Salamé & Baddeley, 1982, 1986, 1989). Especially, it was assumed that speech – even if it is task-irrelevant – gains automatic, obligatory access to the phonological store component of the loop, where it then interferes with the phonological representations of the visually presented list items (“interference-by-content”; Jones & Tremblay, 2000). According to this account, phonological recoding of the visually presented items is a precondition for ISE evocation. However, the phonological loop account was unable to explain later findings demonstrating that (i) the disruptive effect of background speech is independent from its phonological similarity to the list items (e.g., Bell et al., 2011), (ii) nonspeech sounds such as tones (Jones & Macken, 1993) and instrumental music (Schlittmeier et al., 2008) also cause disruption, (iii) the ISE is reliable only with tasks that require serial order retention (Hughes et al., 2007), and (iv) the ISE is determined by the “changing-state”-nature of the sound (Jones et al., 1992). Concerning the latter, serial recall performance is especially impaired by irrelevant sounds with a changing-state characteristic, i.e., by auditory streams consisting of distinct auditory-perceptive objects that vary consecutively. For example, sounds consisting of different syllables or tones evoke an ISE, whereas steady-state sounds, for example, babble noise or repetitions of single syllables or tones, have little or no effect (for an overview, see Schlittmeier et al., 2012). The increase in disruption of serial recall performance evoked by changing-state sounds (e.g., sequences of different syllables) when compared to steady-state sounds (e.g., repetitions of a single syllable) is termed the *changing-state effect* (CSE).

Aiming for a comprehensive account of these findings, Jones and co-authors (Jones et al., 1996, 1999; Jones & Tremblay, 2000) suggested that, for changing-state sounds, the pre-attentive processes of auditory perceptual organization involve seriation of the order of the auditory tokens that make up the irrelevant stream. The resulting order cues interfere with the deliberate seriation processes involved in rehearsing the sequence of memory items, leading to impaired serial recall. In contrast to the phonological loop account, the changing-state account states that it is not the similarity of *contents,* but the similarity of *processes* that leads to disruption (“interference-by-process,” Hughes et al., 2007; Macken, Tremblay, et al., 1999a). In contrast to Baddeley´s multi-component WM model, the changing-state account is based on a non-modular model of serial short-term memory in which events of different origin or modality share a common level of representation (object-oriented episodic memory (O-OER) model, Jones et al., 1996; Jones & Macken, 2018). Rather than postulating domain-specific short-term stores and maintenance mechanisms, the O-OER model follows an embodied approach, and argues that serial short-term memory is a by-product of general-purpose perceptual and motor functions. When serial recall of unconnected items is required (e.g., unrelated words, digits, or spatial positions), these functions are adopted to establish inter-item associations that are necessary for serial order retention. This is achieved by mapping the perceptual input (the to-be-remembered sequence) to a motor output plan (e.g. articulatory in case of verbal items, oculomotor in case of spatial positions), a record of which is then cyclically repeated in support of serial recall. This process is impaired by task-irrelevant changing-state sounds, as they are automatically organized into streams of ordered objects (Bregman, 1994), which then act as competing candidates for motor sequence planning (Hughes & Marsh, 2017; Macken et al., 2015).

According to this view, the disruption of serial recall performance by changing-state sounds is not modulated by the participants’ attention control or focal task engagement. Furthermore, because the changing-state account attributes the disruptive effect to a domain-general rehearsal mechanism, it predicts that irrelevant speech should impair serial recall of both verbal and nonverbal, visuo-spatial items. Data consistent with this assumption of “functional equivalence” were reported by Jones et al. (1995, Exp. 4). They showed that changing-state speech (i.e., sequences of different syllables) evoked comparable impairments in serial order retention of verbal items (visually presented digits) and spatial items (locations of dots on the screen), whereas steady-state speech (i.e., repetitions of a single syllable) had no effect in either task. This finding is at odds with the classical WM model, which assumes separate storage systems and rehearsal mechanisms for visuo-spatial and verbal materials and thus predicts specific interference of irrelevant speech with only the latter (for a thorough discussion, see Meiser & Klauer, 1999).

Taken together, both the phonological loop and the changing-state account attribute the ISE to specific interference between obligatory sound processing and task demands, but they differ considerably with respect to the nature of the assumed sound-induced interference. Neither of these two accounts explicitly specifies a role of attention in the ISE.

The duplex-mechanism framework has been challenged by other authors who prefer a more parsimonious account, arguing that the ISE – and its main characteristic, the CSE – are simply another manifestation of sound-induced attention capture. The auditory deviation effect and the ISE are thus attributed to one and the same mechanism (Bell et al., 2019a; Cowan, 1995, 1999; Elliott, 2002; Körner et al., 2017, 2019). Specifically, in Cowan´s (1995) embedded processes model, working memory is the activated part of long-term memory, and items are kept active in working memory through attentional refreshing. Attentional refreshing is a domain-general maintenance mechanism that allows multimodal representations to be refreshed by bringing them cyclically into the focus of attention. This process is impaired by involuntary diversions of attention towards the task-irrelevant sound.

Different experimental strategies have been used in order to test the contrasting accounts. In a series of studies, Hughes et al. (2005, 2007, 2013) provided evidence for the duplex-mechanism account by confirming the predicted dissociation between the auditory deviation effect and the CSE. In these studies, the CSE is considered instead of the ISE, since the latter may result from the combined action of both mechanisms, i.e., a steady-state effect indexing attention capture (Bell et al., 2019b), and a changing-state effect indexing interference-by-process (AuBuchon et al., 2019). The findings revealed independent, additive effects of changing-state speech and unexpected voice deviants on verbal serial recall performance (Hughes et al., 2007), as well as differential effects of moderating variables: The CSE, but not the deviation effect, was confined to a task requiring serial order retention (Hughes et al., 2007), whereas the deviation effect, but not the CSE, was confined to the encoding phase of the task and was moderated by manipulations of the participants’ attention control, i.e., by providing warnings on the upcoming irrelevant sound, by focal task engagement, and by individual working memory capacity (WMC) (Hughes et al., 2013; Sörqvist, 2010). Other studies indicated dissociable effects of habituation to the auditory deviation effect and the CSE. While habituation to the deviation effect has consistently been reported (for review, see Sörqvist, 2010), little evidence was found for habituation to the disruptive effects of changing-state speech on serial recall (Ellermeier & Zimmer, 1997; Hellbrück et al., 1996; Röer et al., 2011; Tremblay & Jones, 1998).

However, more recent attempts to replicate these findings failed. For example, Körner et al. (2019) found that the deviation effect and the CSE were equally affected by the timing of the auditory distractors in the experimental task. Both effects were most pronounced when the sounds were presented in the second half of the encoding phase, presumably because of the necessity to coordinate rehearsal of already presented items with encoding of the subsequent ones. Other studies found evidence for habituation to changing-state sounds (Pelletier et al., 2016; Röer et al., 2014) and equivalent habituation rates for auditory deviants and changing-state speech (Röer, Bell, Marsh, & Buchner, 2015b). Furthermore, Röer, Bell, and Buchner (2015a) found that the impairment evoked by changing-state speech was attenuated when specific foreknowledge on the upcoming sound was provided and, in two studies with considerable statistical power, the auditory deviation effect and the CSE were equally uncorrelated to the participants´ attention control, measured by WMC (Körner et al., 2017; Röer, Bell, Marsh, & Buchner, 2015b). These findings are at odds with the duplex-mechanism account and are in line with the unitary view that both the CSE and the deviation effect result from a capture of attention.

Another strategy for exploring the role of attention in the ISE has been pursued in studies of potential differences in the ISE between children and adults (Elliott, 2002; Elliott et al., 2016; Elliott & Briganti, 2012; Joseph et al., 2018; Klatte et al., 2010; Meinhardt-Injac et al., 2022; Röer et al., 2018). The rationale behind these studies was that, if the ISE results from sound-induced attention capture, children should be especially prone to disruption due to their underdeveloped attention control. This argument seems reasonable in view of developmental studies on cross-modal selective attention that show that children´s ability to focus on visual categorization tasks in the presence of irrelevant sounds improves continuously during early and middle childhood and is adult-like at about age 10 years (Gumenyuk et al., 2004; Wetzel et al., 2016, 2019). However, the findings concerning developmental change in the effects of irrelevant sounds on serial short-term memory are inconsistent. Some studies showed significantly greater impairments due to changing-state speech in 7- to 10-year-old children when compared to young adults (Elliott, 2002; Elliott & Briganti, 2012, Exp. 3; Elliott et al., 2016, Exp. 1), whereas others found equivalent effects across age groups (Elliott et al., 2016, Exp. 2; Joseph et al., 2018; Klatte et al., 2010; Meinhardt-Injac et al., 2022; Röer et al., 2018; Schwarz et al., 2015). Furthermore, as Elliott et al. (2016) point out, an enhanced ISE in children does not unequivocally confirm the attention capture account, but may also result from increased susceptibility to interference-by-process due to children´s immature, less robust rehearsal skills.

### Research interest

In the current study, we aimed to investigate the roles of attention capture, serial order retention, and phonological processing in the ISE. To this end, we tested the effects of task-irrelevant steady-state and changing-state speech on serial order reconstruction of verbal and spatial items in children and adults. We recruited children aged about 9 years, as we may assume that children of this age use verbal rehearsal when memorizing verbal items (Elliott et al., 2021; Lehmann & Hasselhorn, 2012). As memory items in the verbal and spatial task we used words presented pictorially and locations of dots on the screen, respectively.

Whether or not serial recall of spatial items is impaired by irrelevant speech is still unclear. In view of its theoretical importance, the number of studies addressing this question is surprisingly small. As stated above, Jones et al. (1995) reported equivalent effects of changing-state speech on serial recall of verbal and spatial items. However, later attempts to replicate the findings reported in Jones et al. (1995) yielded inconsistent results (for a partial replication, see Tremblay et al., 2012; for contradicting evidence, see Bergström et al., 2012; Klatte & Hellbrück, 1997; Kvetnaya, 2018).

The predictions for the current study are straightforward. If the ISE results solely from attention capture (unitary view), it should occur irrespective of item type (verbal vs. spatial), and it should be more pronounced in children due to their immature attention control. Furthermore, the disruptive effect of changing-state speech should diminish over the course of the experiment as a result of habituation. In contrast, if the ISE results from specific interference between order information derived from obligatory sound segmentation and deliberate rehearsal (changing-state account), the effect should be evident in both the verbal and the spatial task in both age groups (provided that the children make use of serial rehearsal in the same way as the adults), and remain constant during the course of the experiment. Finally, if phonological processing is a precondition for ISE evocation (phonological loop account), the ISE should be reliable for the verbal task, whereas the spatial task should be unimpaired in both children and adults.

## Methods

### Participants

A total of 83 adults^1^ (students and staff of the University of Kaiserslautern, 50 females, aged between 19 and 32 years), and 81 third-grade children from three primary schools in the Kaiserslautern region (44 females, mean age 8 years, 10 months, SD 9 months) took part in the study. Following prior studies (Bergström et al., 2012; Jones et al., 1995; Klatte & Hellbrück, 1997), we treated sound condition as the within-subjects factor and task as the between-subjects factor. The verbal task was performed by 43 adults (24 females) with a mean age of 23.5 years (*SD* = 2.63 years), and 30 children (19 females) with a mean age of 9 years, 4 months (*SD* = 4 months). The spatial task was performed by 40 adults (26 females) with a mean age of 24.0 years (*SD* = 2.49 years) and 51 children (29 females), with a mean age of 8 years, 7 months (*SD* = 6 months). All participants were native German speakers and had both normal or corrected-to-normal vision and normal hearing according to either self-reports (adults) or parental reports (children). The study was approved by the Rhineland-Palatinate school authority and by the ethics committee of the German Psychological Society (Deutsche Gesellschaft für Psychologie; DGPs). Informed written consent was provided by the adult participants and by the parents of the children. Adults received either course credit or payment for participation (10€). Children received small gifts (writing materials, e.g., erasers, pencils).

### Tasks and materials

#### Apparatus

The experiment was programmed in Python 3.7/PsychoPy 3.1.5 (Peirce, 2007), and controlled by 15.6-in. laptop computers (ProBook 650 G1). Display resolution was 1,920 × 1,080, with a refresh rate of 60 Hz. Sounds were presented via headphones (children: Sennheiser 210; adults: Sennheiser HD650) with a Focusrite Scarlett 2i2 2^nd^ Generation audio-interface.

#### Irrelevant sounds

Consonant-vowel syllables were produced by a trained female speaker in a sound-attenuated laboratory and recorded as .wav files with a sampling rate of 44.1 kHz and a 16-bit resolution. The set comprised the syllables /ba:/, /be:/, /bi:/, /bo:/, /bu:/, /de:/, /ga:/, /gi:/, /go:/, /gu:/, /ka:/, /ke:/, /ki:/, /ko:/, /pa:/, /pe:/, /pi:/, /pu:/, /ta:/, /ti:/, /to:/, /tu:/. The syllables were approximately 500 ms in length. In changing-state trials, a random sequence of the syllables was presented with the restriction that adjacent syllables differed in both consonant and vowel. In steady-state trials, the syllable /ba:/ was repeated. A 200-ms silent interval was introduced between adjacent syllables.

#### Serial order reconstruction tasks

In both the verbal and the spatial task, each trial consisted of a presentation phase, a retention interval, and a recall phase. In the presentation phase, items were presented visually one after another with a presentation duration of 1,500 ms and an interstimulus interval of 500 ms. The final list item was followed by a 5,000-ms retention interval. The onset of the recall phase was signaled by the simultaneous re-presentation of all stimuli. Participants then had to reconstruct the serial order by clicking with the mouse on the items in the order of presentation. Clicking an item changed its shading, indicating that it had been selected. There was no time limit for responding and no possibility of error correction. After selecting the final item, participants were presented with a visual cue to start the next trial by pressing the space bar.

**Fig. 1:**
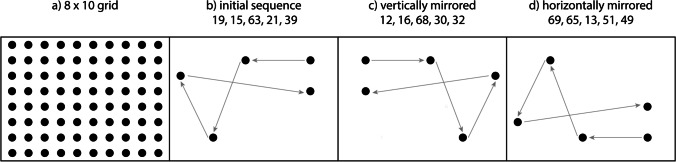
Illustration of the construction of sequences for the spatial task. **a** Total set of locations in an 8 × 10 grid. **b–d** Exemplary five-item sequence with two parallel versions derived by vertical and horizontal mirroring

For the spatial task, stimuli were presented as black dots (10-mm diameter) on a white background in a 304 × 183 mm black frame. Dot locations were drawn from a total of 80 possible locations that were arranged in a 8 × 10 grid (not shown to the participants) in the frame (see Fig. 1a). Prior studies with this task (Parmentier, 2011; Parmentier et al., 2005) have shown that the difficulty of reproducing a sequence depends on the complexity of the spatial path formed by the successive dots, and that path complexity results from three parameters: the number of crossings, the path length, and the angular degree. In order to verify equal task difficulty across sound conditions, we first constructed eight lists of five (children) and 12 lists of seven (adults) dot locations. We then produced two parallel versions of each list by horizontally and vertically mirroring the resulting “path” (for illustration, see Fig. 1b-d). With this strategy, locations differed from trial to trial, but the parameters affecting difficulty were constant for each of the three lists.

For the verbal task, colored drawings representing the monosyllabic German words *Bett, Bus, Eis, Frosch, Kamm, Mond, Pilz, Schal, Schiff*, and *Zaun (bed, bus, ice, frog, comb, moon, mushroom, scarf, ship*, and *fence*) were used for both children and adults. For the adults, the set included the additional items *Brief, Haus, Herz, Hut, Nuss,* and *Schwein (letter, house, heart, hat, nut,* and *pig*). Four lists of five items (drawn out of 10) and six lists of eight items (drawn out of 16) were constructed for the children and adults, respectively. List construction was quasi-random with the restriction that each item occurred in either four lists (children) or six lists (adults). Random permutations of the list items were used to construct two more versions of each list. The drawings were presented in a rectangular black frame (102 × 73 mm) in the center of the white screen. In the recall phase, the pictures were simultaneously re-presented and randomly positioned in a fixed array of five (children) and eight (adults) black frames.

Pictures were used instead of written words to rule out potential impacts of children´s reading ability on performance. Prior studies using pictorial presentation proved detrimental effects of irrelevant speech in children and adults (Klatte et al., 2010), and significant effects of phonological similarity and length of the pictures´ verbal labels in 8-year-old children (Poloczek et al., 2019; Steinbrink & Klatte, 2008), confirming the use of phonological coding and rehearsal. In adults, word length and phonological similarity affect serial order reconstruction of written words and pictorially presented words to the same degree (Schiano & Watkins, 1981), confirming that pictorial presentation does not alter participants´ strategies.

### Procedure

Adults were tested in groups of two to four in a sound-attenuated booth at the University of Kaiserslautern. Children were tested in groups of two to four in a large lecture room in their school. Four computer workplaces were arranged in the room, with a distance of about 10 m between them. Adults received written instruction. Each child was instructed by a researcher or trained student assistant that sat next to the child. In both age groups, the steady- and changing-state sounds were played for 4 s each at the beginning of the experiment, followed by one practice trial per sound condition. All pictures used in the verbal tasks were presented and named by a female speaker. Participants were informed that they should ignore the sounds and focus solely on the serial order reconstruction task.

For both tasks, the three versions of each list were randomly assigned to the sound conditions. Children performed a total of 24 experimental trials, eight per sound condition. Adults completed 16 trials for each sound condition, for a total of 48 experimental trials.

In both groups, sound conditions varied from trial to trial and were quasi-randomized: All sound conditions were presented in random order before being randomized again. In the steady-state and changing-state conditions, the sound started when the participant initiated the trial with the space bar. A random interval between 1,200 and 1,800 ms was introduced before the presentation of the first list item, to avoid correlations between sound and item onset (e.g., Bell et al., 2010). The sound was played throughout both the item presentation and retention interval and stopped at the beginning of the order reconstruction phase. The average sound level (LAeq) of the syllable sequences was 62 dB, as measured by an artificial ear (Brüel & Kjær, Type 4153). The test session lasted about 25 min for children and 35 min for adults.

## Results

**Fig. 2:**
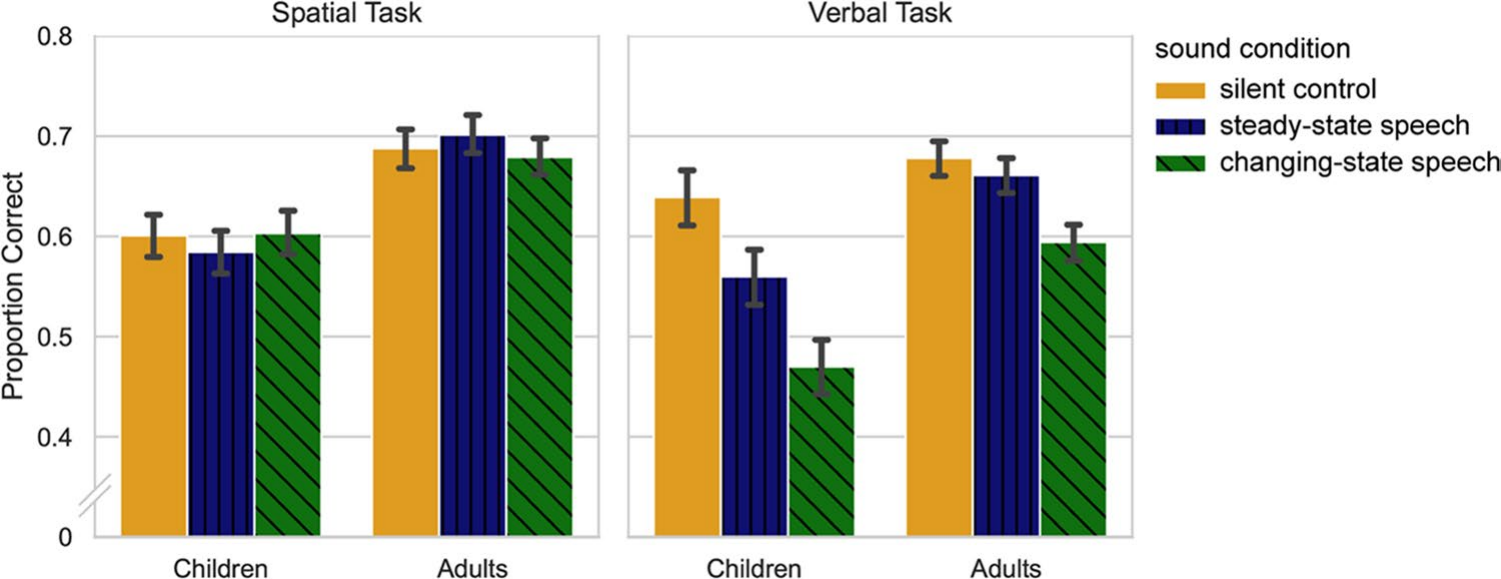
Mean proportion correct scores with respect to task, age group, and sound condition. Error bars denote the bootstrapped confidence intervals

All analyses were based on scores representing the proportion of items recalled at the correct serial position. Proportion correct scores with respect to age group and sound condition are depicted in Fig. 2. Performance in the silent control condition did not differ between age groups in the verbal task (adults: *M* = 0.70, *SD* = 0.15, children: *M* = 0.64, *SD* = 0.16), *t* (71) = 1.61, *p* = .11, but was higher in the adults in the spatial task (adults: *M* = 0.70, *SD* = 0.14, children: *M* = 0.60, *SD* = 0.19), *t* (89) = 2.76, *p* < .01. Performance in the silent control conditions did not differ between tasks, for either the adults or for the children (*t* < 1 in both groups). Thus, a potential age difference in the ISE on the verbal task cannot be attributed to differences in task difficulty between age groups, and a potential difference in the ISE between tasks cannot be attributed to differences in task difficulty within age groups.

The data were analyzed using a 3 × 2 × 2 mixed-design ANOVA with sound condition (silence, steady-state, changing-state) as a within-subject factor and age group (children, adults) and task (verbal, spatial) as between-subjects factors. Greenhouse-Geisser corrections were performed in case of violations of the sphericity assumption according to Mauchly´s test. Power calculations were conducted using G*Power (Faul et al., 2007). The analysis revealed significant main effects of sound condition, *F* (2, 320) = 19.65, *p* < .001, partial *η*^2^ = .11, and age group, *F* (1, 160) = 18.46, *p* < .001, partial *η*^2^ = .10. The main effect of task and the task × age interaction were not significant, *F* (1, 160) = 1.65, *p* = .20, and *F* (1,160) < 1, respectively. Importantly, there was a significant interaction of task × sound condition, *F* (2, 320) = 15.30, *p* < .001, partial *η*^2^ = .09, reflecting a stronger effect of sound on the verbal when compared to the spatial task (see Fig. 2). The sound condition × age group interaction approached significance, *F* (2, 320) = 2.50, *p* = .08, partial *η*^2^ = .02, and there was a significant three-way interaction, *F* (2, 320) = 4.53, p < .05, partial *η*^2^ = .03. In order to explore the significant interactions, separate analyses were performed for the verbal and spatial task.

### Verbal task

The mixed-design ANOVA revealed significant main effects of sound condition, *F* (2, 142) = 38.15, *p* < .001, partial *η2* = .35, and age group, *F* (1,71) = 13.19, *p* < .001, partial *η2* = .16, due to better overall performance in the adults. Furthermore, there was a significant sound × age interaction, *F* (2, 142) = 6.98, *p* < .01, partial *η2* = .09, reflecting stronger sound-induced impairments in the children when compared to the adults. Figure 2 indicates that, in children, both changing-state and steady-state speech evoked a stronger impairment relative to quiet, whereas the difference between the steady-state and the changing-state conditions (i.e., the CSE) is comparable between the age groups. This impression was confirmed statistically: When the silent control condition was dropped from the analysis, the age group × sound interaction proved non-significant, *F* (1, 71) = 2.50, *p* = .12. A power analysis confirmed that, with a total size of N = 73, α = .05, 1 - ß = .80, and a correlation between repeated measurements of r = .70 (calculated from the data), an age × sound interaction of effect size f = .12 could be detected.

The sound × age interaction was further explored by separate analyses in both age groups, which confirmed a significant effect of sound condition in the adults, *F* (2, 84) = 13.65, *p* < .001, partial *η2* = .25, and in the children, *F* (2, 58) = 20.77, *p* < .001, partial *η2* = .42). Bonferroni-corrected post hoc tests revealed that, in the adults, performance in the changing-state condition was lower when compared to steady-state and silence (both *ps* < .001), which did not differ (*p* = .93). In the children, performance with changing-state speech was lower when compared to silence and steady-state speech (both *ps* < .001), and performance in the steady-state condition was lower when compared to silence (*p* < .05). Thus, a steady-state effect was evident in the children, but not in the adults.

In a study with 7- to 8-year-old children, AuBuchon et al. (2019) found that the ISE increased with short-term memory span assessed through a digit span test performed in silence. Especially, children´s digit span explained 29% of variance in the disruption evoked by changing-state speech. This finding is not in line with the view that underdeveloped rehearsal skills make children especially vulnerable to the ISE. In contrast, the ISE in children around 7 years of age seems to *increase* with their rehearsal abilities. Respective studies with adults found no evidence for an association between short-term memory capacity and the ISE (Ellermeier & Zimmer, 1997; Elliott & Cowan, 2005, Exp,1; Elliott et al., 2020), with the exception of Elliott and Cowan (2005, Exp. 2), who reported a significant correlation. Following these studies, we used serial recall performance in quiet as an estimate of individual short-term memory capacity, and difference scores (performance in quiet – performance with changing-state speech) as a measure of ISE (AuBuchon et al., 2019; Ellermeier & Zimmer, 1997). Both measures were normally distributed in children and adults. Replicating AuBuchon et al. (2019), we found that high-performing children exhibited stronger disruption when compared to low performers (*r* = .63, *p* < .001). A similar but weaker association was evident in the adults (*r* = .35, *p* = .05).

**Fig. 3:**
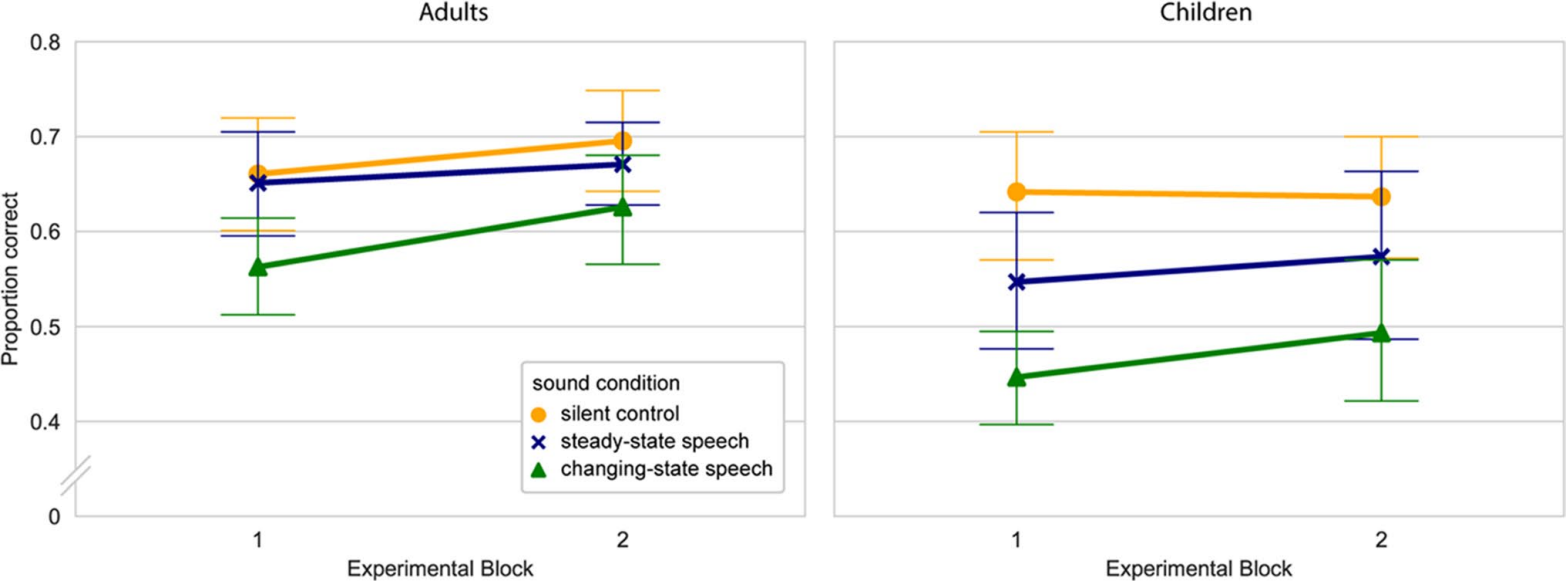
Mean proportion correct scores in the verbal task in two consecutive blocks of four trials each. Error bars denote the bootstrapped confidence intervals

Addressing the role of habituation in the ISE, a further analysis was run to find out whether the impairment decreases over the course of the experiment. For this analysis, the data of the adults were trimmed to the first eight trials per sound condition, in order to match the number of trials included between age groups. For each sound condition, proportion correct scores were calculated for two consecutive blocks of four trials each. Means and standard errors are depicted in Fig. 3. A 3 (sound condition) × 2 (block) × 2 (age group) ANOVA confirmed significant main effects of sound condition, *F* (1, 71) = 29.76, *p* < .001, partial *η*^2^ = .30 and age group, *F* (1, 71) = 7.50, *p* = .01, partial *η*^2^ = .10, and a significant sound condition × age interaction, *F* (1, 71) = 3.54, *p* < .05, partial *η*^2^ = .05. The main effect of block approached significance, *F* (1,71) = 3.64, *p* = .06., partial *η*^2^ = .05, but the sound condition × block interaction, the block × age interaction, and the three-way interaction were not significant (all *F*s < 1). The lack of a sound × block-interaction was confirmed in a further analysis on the full data from the adults, using four consecutive blocks of four trials each. This analysis yielded significant main effects of sound condition, *F* (2,84) = 13.65, *p* < .001, partial *η*^2^ = 0.25, and block, *F* (3, 126) = 7.84, *p* < .001, partial *η*^2^ = .16 but no interaction, *F* (6, 252 < 1. Thus, there was no sign of habituation to irrelevant speech found, neither in the children nor in the adults.

### Spatial task

The mixed-design ANOVA revealed a significant main effect of age group, *F* (1, 89) = 7.30, *p* <.01, partial *η*^2^ = .08, resulting from better overall performance in the adults. The main effect of sound condition and the sound × age interaction were not significant (*F* (2, 178) < 1 in both cases). A power analysis confirmed that, with a total size of n = 91, α = .05, 1 - ß =.80 and a correlation between repeated measurements of r = .75 (estimated from the data), a main effect of sound condition and an age × sound - interaction of effect size f = 0.09 could be detected.

In summary, in the spatial task, there was no evidence for a disruptive effect of changing-state speech when compared to silence, and no evidence for a changing-state effect (i.e., better performance in steady-state when compared to changing-state speech) for either age group.

## Discussion

With the aim of further exploring the role of attention capture, serial order retention, and phonological coding in the ISE, the effects of steady-state and changing-state irrelevant speech (CV-syllables) on serial recall of verbal and spatial items were assessed in third-grade children and adults. The tasks required serial order reconstruction of words presented pictorially (verbal task) and locations of dots on the screen (spatial task).

In the verbal task, both age groups exhibited performance decrements under changing-state speech when compared to silence (ISE), and when compared to steady-state speech (CSE). The ISE was stronger in children, but the magnitude of the CSE did not differ between age groups. Only the children were significantly impaired by steady-state speech. In both age groups, the ISE increased significantly with increasing performance in the silent control condition. The disruption evoked by changing-state speech did not diminish over the course of the experiment, neither in the children nor in the adults. In the spatial task, no evidence for a disruptive effect of irrelevant speech was found, in either the children or the adults.

The immunity of spatial serial recall to disruption through changing-state speech is difficult to reconcile with both the changing-state account and the attentional account of the ISE. According to the changing-state account, the ISE results from pre-attentive processing of order in changing-state sounds, which interferes with the deliberate serial rehearsal of the to-be-remembered sequence. The changing-state account is based on a unitary model of serial short-term memory, assuming that sequences of unrelated items – irrespective of their sensory modality or representational code – share a common maintenance mechanism, i.e., generating and cyclically repeating a record of a motoric reconstruction of the to-be-remembered list. Accordingly, the ISE and the CSE should occur irrespective of item modality (e.g., visual, auditory) or code (e.g., verbal, spatial). Supporting evidence for the latter was provided in Jones et al. (1995, Exp. 4), who reported equivalent effects of irrelevant speech on serial recall of verbal and spatial items. In both tasks, performance was impaired by changing-state speech, but not by steady-state speech. This finding is crucial for the changing-state account of the ISE. However, in the current study, no detrimental effect of changing-state speech was found in the spatial task, whereas in the verbal task, the ISE and CSE were clearly replicated. Prior studies also reported null effects of changing-state speech on spatial serial recall (Bergström et al., 2012; Klatte & Hellbrück, 1997; Kvetnaya, 2018). These failures to replicate the Jones et al. (1995, Exp. 4) finding cannot be attributed to methodological differences or a lack of statistical power, as the tasks and sounds were rather similar, and the numbers of participants were higher when compared to the original study. We cannot provide a clear explanation for the non-replicability of the original finding, but we may conclude that, if an ISE on spatial serial recall exists at all, it differs vastly from the ISE on verbal tasks with respect to robustness and replicability. This is clearly not in line with the claim of “functional equivalence.”

The attention-capture account attributes the ISE to an impairment of the attentional refreshing of the list items due to involuntary diversions of attention towards the task-irrelevant auditory stimuli. Attentional refreshing means that multimodal representations are kept active in working memory by repeatedly drawing them into the focus of attention (Cowan, 1995). As attentional refreshing is a domain-general maintenance mechanism, the attention-capture account predicts an ISE for both the verbal and the spatial task. Furthermore, children should be more impaired than adults because of their immature attention control. In fact, according to the attentional capture account, the sound-induced impairment should be even greater in the spatial when compared to the verbal task in both age groups. There is considerable evidence that visuo-spatial short-term memory tasks – including those requiring serial order retention – rely more than their verbal equivalents on domain-general attentional resources in adults (Morey & Miron, 2016; for review, see Morey, 2018) and children (Alloway et al., 2006; Campos et al., 2013; Michalczyk et al., 2013). Thus, visuo-spatial tasks should be reliably disrupted when attentional resources are bound by task-irrelevant sounds. In line with this, it has been shown that serial spatial recall is susceptible to attention capture through auditory deviants (Morey & Miron, 2016). The null effect of irrelevant speech found in the current study adds to the evidence that the ISE and the auditory deviation effect are dissociable. A further problem for the attentional account is the absence of habituation to the ISE in the verbal task.

In regard to developmental effects, the current study replicated the disruptive impact of changing-state speech on serial recall of verbal items in both age groups, the children were more affected than the adults, and only the children were significantly impaired by steady-state speech. Similar results have been reported by Elliott (2002), Elliott and Briganti (2012, Exp. 3), and Elliott et al. (2016). In the latter study, in addition to serial recall, a further task was included that did not require serial order retention (missing item task, Hughes et al., 2007). The effect of changing-state speech on serial recall was more pronounced in children (Exp. 1), and children but not adults were significantly impaired by both steady-state and changing-state speech in the non-serial task (Exp. 2). The authors discussed these age effects in the framework of the duplex mechanism account, arguing that children and adults are equally prone to interference-by-process, but children are more susceptible to attentional diversions by task-irrelevant sounds, regardless of whether or not they change over time.

Thus, in these studies (Elliott, 2002; Elliott & Briganti, 2012, Exp. 3; Elliott et al., 2016), the enhanced effect of irrelevant speech on serial recall in children has been attributed to children´s increased susceptibility to sound-induced attention capture. This argument is difficult to reconcile with the current finding that serial recall of spatial items was unimpaired by irrelevant speech in children and adults. If irrelevant speech captures children´s attention, it should impair performance for both verbal and spatial items.

Taken together, the finding that irrelevant speech impairs verbal, but not spatial serial recall contradicts the assumption that the ISE results from interference with a domain-general maintenance mechanism, i.e., attentional refreshing (as proposed by the attention-capture account), or serial rehearsal through perceptual-motor sequence planning (as proposed by the changing-state account). Instead, the current results indicate that irrelevant speech interferes with mechanisms or memory traces that are *specific* to the verbal domain, and that children are more susceptible to this kind of interference than adults. Potential candidates as points of attack for irrelevant speech are therefore the efficiency of articulatory rehearsal, and/or the integrity of phonological representations.

Articulatory rehearsal is the repetitive subvocal pronunciations of verbal list items in a sequential manner, using articulatory motor programs that are also involved in language production. Articulatory rehearsal is regarded as the dominant maintenance strategy in verbal serial recall tasks, and is integrated in modular models of working memory as a mechanism specifically dedicated to the prevention of time-based decay of phonological representations within a phonological store (e.g., Baddeley, 2012; Camos et al., 2009). In view of the evidence that the ISE is most pronounced with verbal tasks that require serial order retention (Beaman & Jones, 1997; Elliott et al., 2016; for review, see Hughes et al., 2007), or at least suggest a serial rehearsal strategy (Hughes & Marsh, 2020), articulatory rehearsal seems to be a promising route of entry for irrelevant speech. However, explaining the ISE through specific interference with articulatory rehearsal is not without problems. First, there is evidence that cumulative articulatory rehearsal does not improve serial recall performance in adults (Souza & Oberauer, 2018), and that children below the age of 10 years employ various strategies in visual-verbal serial recall tasks (Koppenol-Gonzalez et al., 2014), with cumulative rehearsal being not the dominant one (Poloczek et al., 2019). It is difficult to explain how the disruption of a strategy that is inefficient or rarely used should lead to a performance decrement. Second, in line with this argument, preventing articulatory rehearsal through articulatory suppression (i.e., continuously uttering an irrelevant syllable) during the retention interval (Toppino & Pisegna, 2005), or rapid presentation of the visual list items (AuBuchon et al., 2020; Samper et al., 2021) does not diminish the magnitude of the ISE. Third, the rehearsal account of the ISE is mainly based on findings that non-serial tasks are not or less affected by irrelevant speech. However, the non-serial tasks used in the respective studies, i.e., free recall (Beaman & Jones, 1998; Salamé & Baddeley, 1990) and the missing item task (e.g., Beaman & Jones, 1997; Elliott et al., 2016; Hughes et al., 2007), rely heavily on semantic strategies and thus differ from serial recall not only with respect to serial order retention, but also with respect to the role of phonological processing. It has been shown that working memory tasks that do not involve serial rehearsal (or at least strongly discourage its use), but require phonological processing, i.e., the maintenance, analysis, or manipulation of phonological representations, are reliably affected by irrelevant speech in children (Klatte et al., 2007) and adults (Klatte et al., 2019; Samper et al., 2021; Smith et al., 1995). These findings open the possibility that it is not the serial order component, but the reliance on phonological representations that makes verbal serial recall vulnerable to disruption by irrelevant speech.

Attributing the ISE to a corruption of phonological traces might also explain the differences between children and adults with respect to the magnitude of the ISE, and its relation to overall serial recall performance. Children might be more affected by irrelevant speech than adults because their phonological representations are less well specified (Claessen et al., 2009; Hazan & Barrett, 2000; Metsala & Walley, 1998), and therefore more vulnerable to disruption. The correlation of performance in the silent control condition and the ISE magnitude might be explained by assuming that high performers rely more than low performers on phonological strategies (Poloczek et al., 2019), making them more prone to disruption by irrelevant speech.

The view that irrelevant speech evokes “interference-by-content” in that it impairs phonological codes has originally been taken by the phonological loop account of the ISE (Salamé & Baddeley, 1982), and was later adopted and mathematically specified in the feature model of working memory (Neath, 2000). In the latter, items are represented by vectors of modality-dependent and modality-independent features. Modality-dependent features are generated during encoding and represent raw, physical aspects of the stimuli. Modality-independent features are produced through internal processes such as identification and categorization. The detrimental effect of irrelevant speech is modelled by replacing modality-independent features of the list items by similar modality-independent features of the irrelevant speech (“feature adoption”). These accounts were more or less abandoned, mainly because of studies showing that the overlap of phonemes in the irrelevant speech tokens and the list items (“between-stream-similarity”) does not affect the magnitude of the disruption (Bell et al., 2011; Hughes et al., 2005; Larsen et al., 2000; LeCompte & Shaibe, 1997), and that non-speech sounds with a changing-state characteristic, such as tone sequences or instrumental music, also disrupt verbal serial recall (Jones & Macken, 1993; Schlittmeier et al., 2008).

However, to reject the assumption of content-based interference might have been premature, given the evidence that supports a role of similarity in the disruption of serial recall performance by irrelevant sounds. Specifically, it has been shown that speech or speech-like sounds are more disruptive to verbal serial recall than non-speech sounds (Schlittmeier et al., 2012), even with acoustic complexity controlled (Dorsi et al., 2018; Viswanathan et al., 2014), that the magnitude of the ISE depends on the overlap of phonetic features (though not of the phonemes) in the irrelevant speech tokens and the list items (Eagan & Chein, 2012), and that speech interferes more with the serial order retention of verbal when compared to tonal items, whereas irrelevant tones interfere more with the retention of tonal when compared to verbal sequences (Defilippi et al., 2019; Kattner & Meinhardt, 2020; Williamson et al., 2010). These findings suggest an important role of between-stream-similarity in the disruptive effects or irrelevant sounds, and thus content-based interference.

To summarize, the current study showed that the disruptive effect of irrelevant speech on serial recall performance is confined to the verbal domain, and is more pronounced in children compared to adults. These results are difficult to explain with the dominant accounts of the ISE, i.e., specific interference with serial order retention, or attention capture. We argue that phonological processing, but not serial order retention is a precondition for ISE evocation, and that irrelevant speech impairs verbal serial recall and other tasks that rely on phonological processing by corrupting phonological traces in working memory.

This study is limited, as it does not include nonspeech sounds nor phonological processing tasks that do not require serial order retention. Furthermore, the current findings do not allow a clear specification of the mechanism that evokes the proposed interference with phonological codes. Feature-based models of working memory may provide a parsimonious account of similarity-based interference effects, as they are able to explain specific interference without postulating specialized working memory modules (Morey, 2018). It has already been shown that Nairne´s feature model (Nairne, 1990; Neath, 2000) successfully simulates specific interference through verbal and spatial secondary tasks on verbal and spatial working memory (Poirier et al., 2019). As pointed out by Eagan and Chein (2012), with some modifications, the feature model could also account for the effects of irrelevant sounds, and their dependencies on participants´ individual strategies.

## Data Availability

The data and materials are available at: https://osf.io/pyqvr/

## Declarations

In the first version of this manuscript, we reported the data from a different sample of adults (N = 80). These participants were tested with two further sound conditions, i.e., five sound conditions in total. During the review process, it became clear that the imbalance in sound conditions makes valid comparisons between the two age groups difficult. Therefore, we recruited a new sample of adults (N = 83) in early 2022, and used only the same three sound conditions that were presented to the children. The pattern of results was perfectly replicated with the new sample. Here, only the data from the new sample are reported.
